# Procyanidin C1 from *Viola odorata* L. inhibits Na^+^,K^+^-ATPase

**DOI:** 10.1038/s41598-022-11086-y

**Published:** 2022-04-29

**Authors:** Tomas Heger, Marek Zatloukal, Martin Kubala, Miroslav Strnad, Jiri Gruz

**Affiliations:** 1 Department of Experimental Biology, Faculty of Science, Palacky University, Olomouc, Czech Republic; 2 Department of Chemical Biology, Faculty of Science, Palacky University, Olomouc, Czech Republic; 3 Department of Experimental Physics, Faculty of Science, Palacky University, Olomouc, Czech Republic; 4 Laboratory of Growth Regulators, Institute of Experimental Botany of the Czech Academy of Sciences, Palacky University, Olomouc, Czech Republic

**Keywords:** Chemical biology, Plant sciences

## Abstract

Members of the *Viola* genus play important roles in traditional Asian herbal medicine. This study investigates the ability of *Viola odorata* L. extracts to inhibit Na^+^,K^+^-ATPase, an essential animal enzyme responsible for membrane potential maintenance. The root extract of *V. odorata* strongly inhibited Na^+^,K^+^-ATPase, while leaf and seeds extracts were basically inactive. A UHPLC-QTOF-MS/MS metabolomic approach was used to identify the chemical principle of the root extract’s activity, resulting in the detection of 35,292 features. Candidate active compounds were selected by correlating feature area with inhibitory activity in 14 isolated fractions. This yielded a set of 15 candidate compounds, of which 14 were preliminarily identified as procyanidins. Commercially available procyanidins (B1, B2, B3 and C1) were therefore purchased and their ability to inhibit Na^+^,K^+^-ATPase was investigated. Dimeric procyanidins B1, B2 and B3 were found to be inactive, but the trimeric procyanidin C1 strongly inhibited Na^+^,K^+^-ATPase with an IC_50_ of 4.5 µM. This newly discovered inhibitor was docked into crystal structures mimicking the Na_3_E_1_∼P·ADP and K_2_E_2_·P_i_ states to identify potential interaction sites within Na^+^,K^+^-ATPase. Possible binding mechanisms and the principle responsible for the observed root extract activity are discussed.

## Introduction

Na^+^,K^+^-ATPase (NKA; EC 7.2.2.13) is a transmembrane enzyme found in every animal cell. It is responsible for maintaining the plasma membrane potential and sodium gradient necessary for the function of many secondary active transporters^1,2^. Its importance in clinical practice is demonstrated by the frequent prescription of NKA inhibitors such as digoxin, which is used against cardiac insufficiency^3^. Moreover, rare mutations of NKA cause severe diseases including familial hemiplegic migraine type 2 and rapid-onset dystonia-parkinsonism^4,5^. Two hundred and thirty-six years after the first publication of William Withering’s book *An Account of the Foxglove, and Some of Its Medical Uses*, which describes medical uses of digitalis, the pharmacology of NKA remains interesting but incompletely understood. In addition to its role in heart failure management, recent high-throughput screening efforts suggest that NKA inhibition may have positive effects on hypercholesterolemia, several types of cancer, and viral infections^6–15^.

NKA consists of a ~ 112 kDa catalytic α subunit with ten transmembrane segments, and a ~ 55 kDa glycosylated β subunit with one transmembrane segment and three isoforms (β_1-3_) that acts as a chaperone and modulatory protein^1,16,17^. The catalytically active αβ heterodimer can also associate with one of the regulatory and stabilizing FXYD proteins^18,19^.

The catalytic cycle of NKA involves alternating-access ion transport and a series of large conformational changes^20–22^. As a translocase, NKA hydrolyzes ATP to transfer three Na^+^ ions to the extracellular space and two K^+^ ions to the cytoplasm^23^. The resulting ion gradient across the plasma membrane is central to the physiology of excitable cells.

Other important functions of NKA include thermogenesis^24^ and promotion of cell adhesion and interaction via its β_1_ subunit^25–27^. It was also shown that the β_1_ subunit can act as a general K^+^-dependent lectin^28^, and that its expression suppresses the motility and invasiveness of carcinoma cells^29^. In addition to these functions, NKA is part of a complex signaling network that is currently under investigation.

In signal transduction pathways, NKA acts as a receptor for cardiotonic steroids (CTSs), which are highly selective inhibitors that bind in a cavity accessible from the extracellular side^30^. Many studies have shown that upon CTS binding, NKA interacts with several downstream proteins via its intracellular side and triggers a signaling cascade^31,32^. The first report describing an endogenous CTS was published in 1991^33^, and endogenous CTS are now regarded as a distinct class of hormones^34,35^. However, uncertainties remain concerning their biosynthesis and distribution^36,37^.

*Viola* is the largest genus of the *Violaceae* family, containing up to 600 species^38^. Members of this genus are used in traditional herbal medicine in many regions: *V. odorata* is used in Pakistan^39^, *V. betonicifolia* and *V. canescens* in the Himalayas^40,41^, *V. hondoensis* in Korea^42^, and *V. yedoensis*, *V. kunawarensis*, and *V. tianschanica* in Central and East Asia^43,44^.

Sweet violet (*Viola odorata*) is traditionally used against several diseases, including neurological disorders and hypertension^45^. Volatile components of *V. odorata* leaf extracts include nona-2,6-dienal and (*Z*)-hex-3-enal, which are the compounds primarily responsible for its odor. Most of the other volatile components are saturated or unsaturated aliphatic hydrocarbons and related oxidized species^46^. Another study identified phthalate, tetrahydrobenzofuranone derivative, and monoterpenoids as the main components of leaf extracts of an Iranian chemotype of *V. odorata*^47^. In addition, cyclic peptides called cyclotides (e.g. cycloviolacins) with diverse biological activities are found in various sweet violet organs^48,49^. The phytochemistry of the related species *V. betonicifolia* has been reviewed in detail^50^.

There is relatively little published information on the secondary metabolite content of root extracts of *V. odorata*, which are the subject of this work. Phenolic acids and the flavonoid compounds vitexin and rutin were quantified in the roots of *V. tricolor* during a study on mycorrhizal colonization that demonstrated the influence of mycorrhizal fungi on secondary metabolite levels^51^. Similar endophyte-induced changes in phytochemical composition are well documented^52,53^. In *V. odorata*, the diversity of endophytes in the roots was higher than in other organs^54^.

Procyanidins are polyphenolic compounds consisting of condensed flavan-3-ol subunits. Monomers of (–)-epicatechin or ( +)-catechin form oligomeric or polymeric structures that are classified based on the nature of their interflavan bonds. Specifically, they are categorized based on the stereochemistry of the interflavan bond (α or β) and the atoms linked by the bond: bonds between atoms C-4 and C-8 or C-4 and C-6 are classified as B-type linkages, while those between C-4 and C-8 along with C-2 → O → C-7 ether bond are A-type linkages^55^.

Here, we investigate the NKA-inhibiting activity of *Viola odorata* L. extracts and their components. Active compounds are identified by LC–MS correlation metabolomics^56^.

## Results

**Figure 1:**
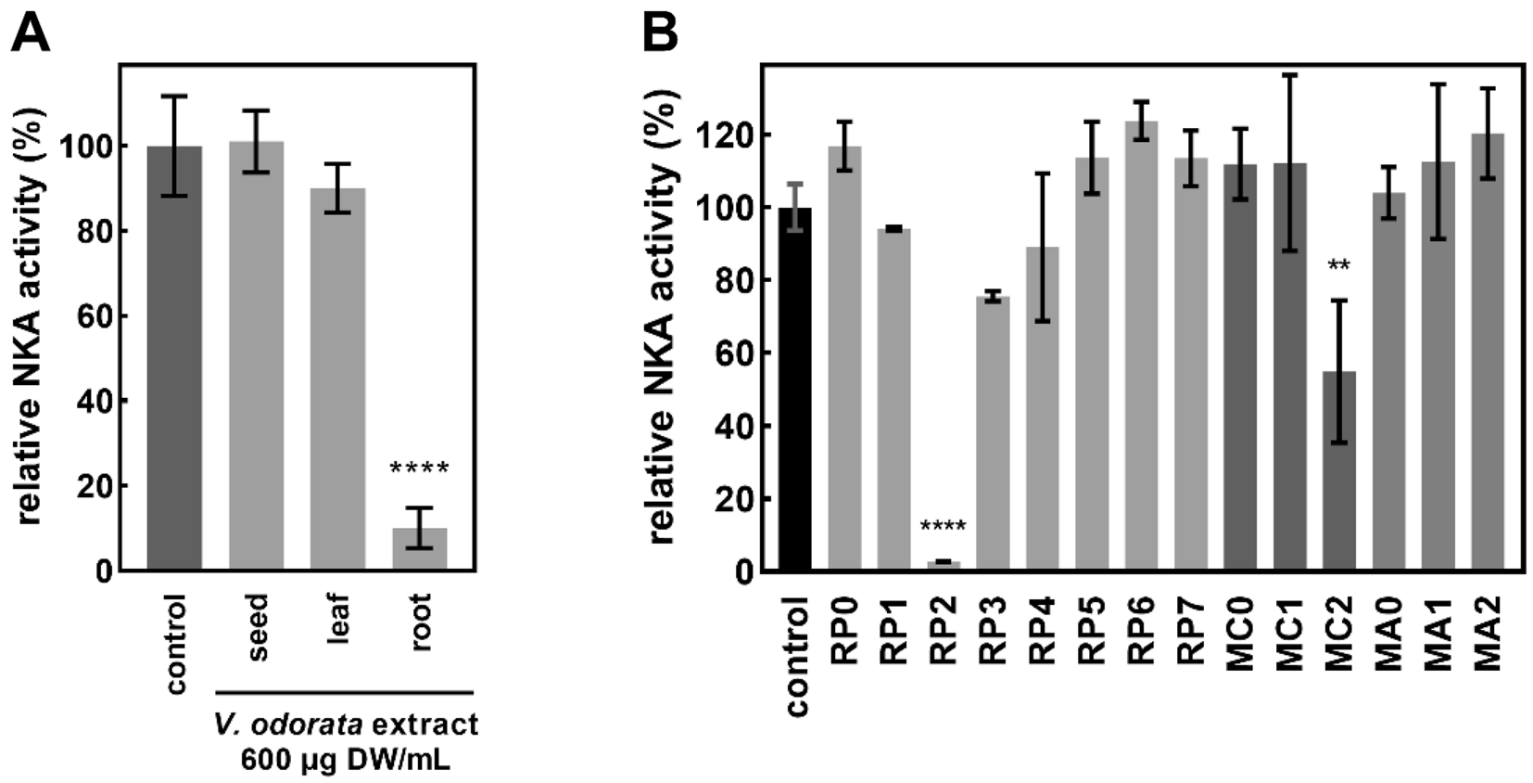
(**A**) Relative NKA activity (%) of Na^+^,K^+^-ATPase treated with crude methanolic *Viola odorata* extracts. Data are expressed as means ± SD (n = 3–4); Statistical significance based on Tukey’s test is indicated by **** for *p* < 0.0001. (**B**) Relative NKA activity (%) of Na^+^,K^+^-ATPase treated with *V. odorata* root extract fractions. Data are expressed as means ± SD (n = 3–4); Statistical significances based on Tukey’s test are indicated by **** and ** for *p* < 0.0001 and for *p* < 0.005, respectively.

Over 50 plant species were extracted and screened for NKA inhibiting activity (Supplementary Table S1 online). Individual parts of the most promising candidate, *Viola odorata* L., were then analyzed separately to evaluate their activity (Fig. 1A). Interestingly, although seed and leaf extracts showed no significant activity, root extracts inhibited NKA by over 90% at a concentration of 600 µg DW/mL. Therefore, the root extract was further investigated by separating it into fractions using C18, mixed-mode anion, and cation exchange SPE columns. This separatory process yielded 14 fractions, of which two (RP2 and MC2) were significantly active (Fig. 1B). A recently published correlation-metabolomic approach was then applied to these fractions to identify candidate features possibly responsible for the observed NKA-inhibiting activity^56^.

**Figure 2:**
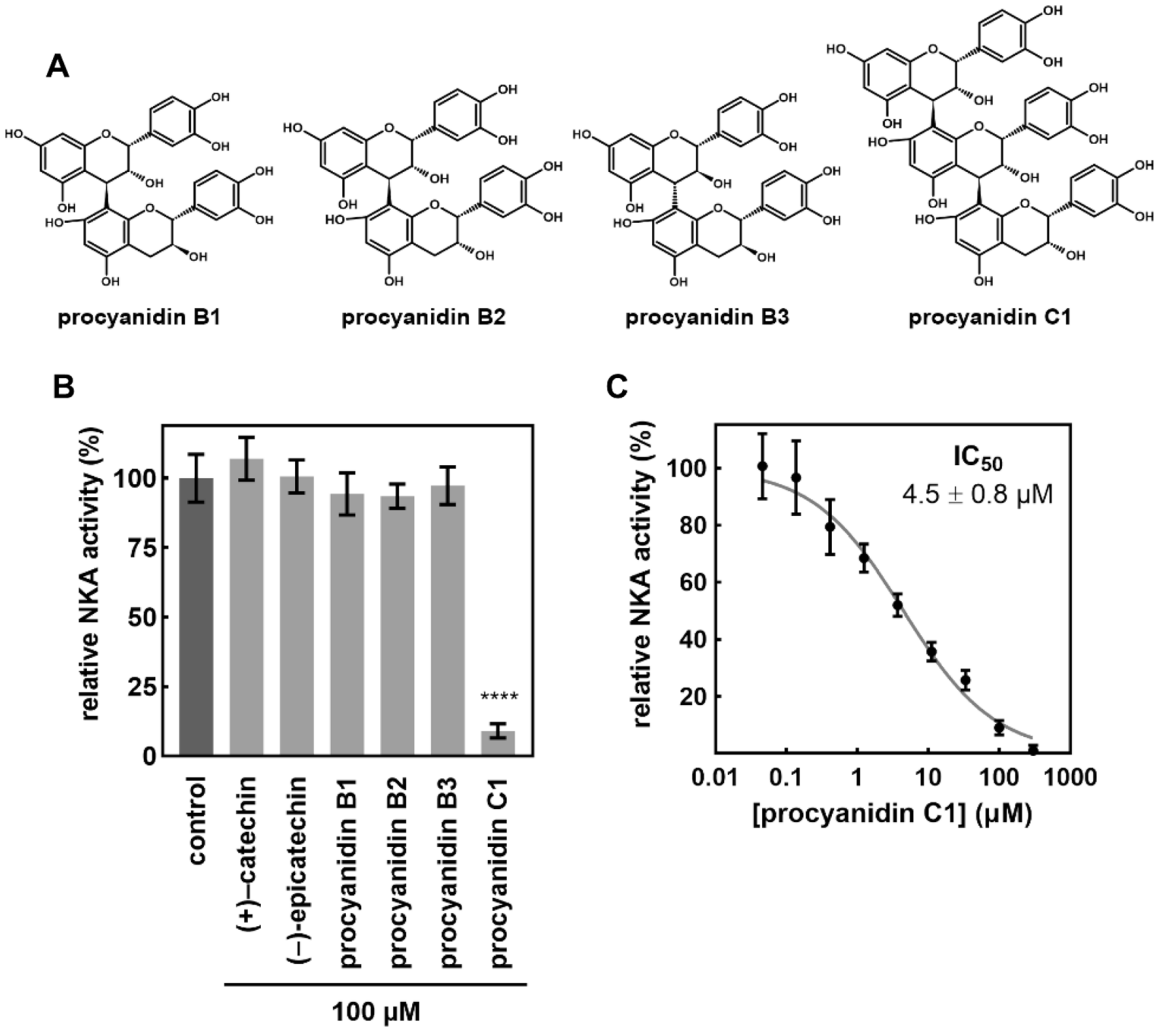
(**A**) Chemical structures of tested procyanidins. (**B**) Relative NKA activity (%) of NKA treated with flavan-3-ols and B-type procyanidin dimers and trimer. Data are expressed as means ± SD (n = 4). Statistical significance based on Tukey’s test is indicated by **** for *p* < 0.0001. (**C**) Dose–response curve for NKA inhibition by procyanidin C1. Data are expressed as means ± SD (n = 8).

All isolated fractions were analyzed by non-targeted UHPLC-QTOF-MS/MS, which revealed 15,331 features in negative and 19,961 features in positive ion modes. Combined features from both modes were filtered based on their abundance and retention time, resulting in 2,748 features in total. These features were correlated with the inhibitory activity of each fraction and sorted according to the calculated correlation coefficients. The dimensionality of the data was further reduced by manual identification of the molecular ions of adducts, fragments, and isotopic peaks. Data from positive and negative modes were processed separately, which typically resulted in the presence of two rows independently referring to the same metabolite (see for example rows 1 and 2 in Table 1). The highly correlated candidate metabolites are shown in Table 1. Briefly, the higher the r value, the greater the likelihood that the row represents the active component. The majority of the candidate metabolites were annotated as B-type procyanidins, with the exception of one that was putatively identified as feruloyl putrescine. The commercially available procyanidins B1, B2, B3 and C1 were purchased and analyzed, allowing two candidates to be unambiguously identified as procyanidins C1 and B2. The NKA inhibitory activities of all available procyanidins (B1, B2, B3 and C1) were also determined (Fig. 2A; 2B), revealing that procyanidin C1 is a strong inhibitor with an IC_50_ of 4.5 ± 0.8 µM (Fig. 2C).

**Table 1:** Top 15 candidate features from the metabolomic analysis of *Viola odorata* root fractions based on the Pearson correlation coefficient r. Metabolites were putatively identified (annotated) by analyzing their MS^2^ spectra with the exception of procyanidins B2 and C1, which were identified by direct comparison with authentic standards.

No.	Measured *m/z*	Theoretical *m/z*	Mode	Δppm	RT (min)	r	Annotation
1	867.2136	867.2136	pos	0.0	5.89	0.9599	B-type procyanidin trimer 1
2	865.1971	865.1980	neg	–1.0	5.88	0.9581	B-type procyanidin trimer 1
3	577.1348	577.1346	neg	0.3	7.61	0.9562	B-type procyanidin dimer
4	579.1508	579.1503	pos	0.9	7.61	0.9540	B-type procyanidin dimer
5	867.2133	867.2136	pos	–0.3	7.12	0.9474	B-type procyanidin trimer 2
6	579.1501	579.1503	pos	–0.3	6.33	0.9463	Procyanidin B2
7	1155.2785	1155.2614	pos	0.2	5.86	0.9403	B-type procyanidin tetramer
8	865.196	865.1980	neg	–2.3	7.09	0.9392	B-type procyanidin trimer 2
9	577.1346	577.1346	neg	0.0	6.33	0.9367	Procyanidin B2
10	867.2137	867.2136	pos	0.1	7.25	0.9343	Procyanidin C1
11	1153.2585	1153.2614	neg	–2.5	5.86	0.9324	B-type procyanidin tetramer
12	865.1966	865.1980	neg	–1.6	7.24	0.9306	Procyanidin C1
13	867.2137	867.2136	pos	0.1	5.74	0.9253	B-type procyanidin trimer 3
14	265.1551	265.1552	pos	–0.4	5.00	0.9196	Feruloyl putrescine
15	865.1977	865.1980	neg	–0.3	7.72	0.9118	B-type procyanidin trimer 4

**Figure 3:**
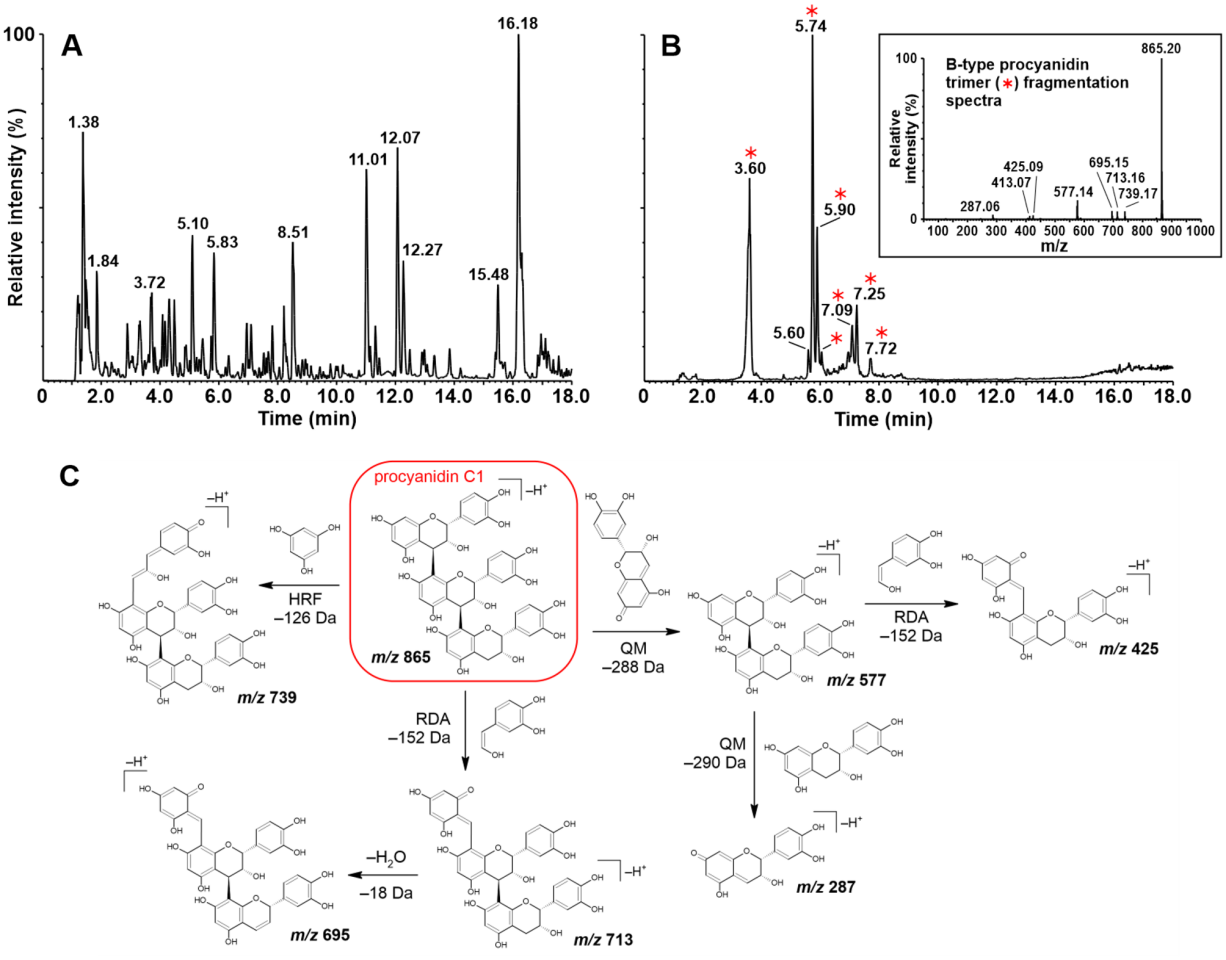
(**A**) UHPLC-ESI–MS base peak chromatogram of a crude *Viola odorata* root extract (160 mg DW/mL) recorded in negative ion mode. (**B**) Extracted ion chromatogram recorded in negative ion mode (*m/z* 865). Analytes showing fragmentation patterns characteristic of B-type procyanidin trimers are indicated by red asterisks. Inset: B-type procyanidin trimer fragmentation spectrum obtained in negative ion mode with a collision energy of 20 eV. (**C**) B-type procyanidin trimer fragmentation pathways of procyanidin C1^57^; QM quinone methide cleavage; HRF heterocyclic ring fission; RDA retro-Diels–Alder cleavage.

Due to the complexity of the *V. odorata* extracts, it was not possible to structurally identify the other B-type procyanidins from the candidate list. The diversity of B-type procyanidins in *Viola odorata* root is illustrated by the number of peaks with *m/z* 865 showing fragmentation patterns characteristic of B-type procyanidin trimers (Fig. 3). The procyanidin C1 (7.25 min) is a minor peak among detected procyanidin trimers in the *V. odorata* extract (Fig. 3B).

Molecular docking of procyanidin C1 to NKA was performed to identify possible interaction sites. Two crystal structures of NKA representing different catalytic intermediates were selected for the docking study: 3WGU (Na_3_E_1_[AlF_4_]^–^·ADP), which has a resolution of 2.80 Å and is an analogue of the Na_3_E_1_∼P·ADP state, and 2ZXE (K_2_E_2_·[MgF_4_]^2–^), which has a resolution of 2.40 Å and is analogous to the K_2_E_2_·P_i_ state.

**Figure 4:**
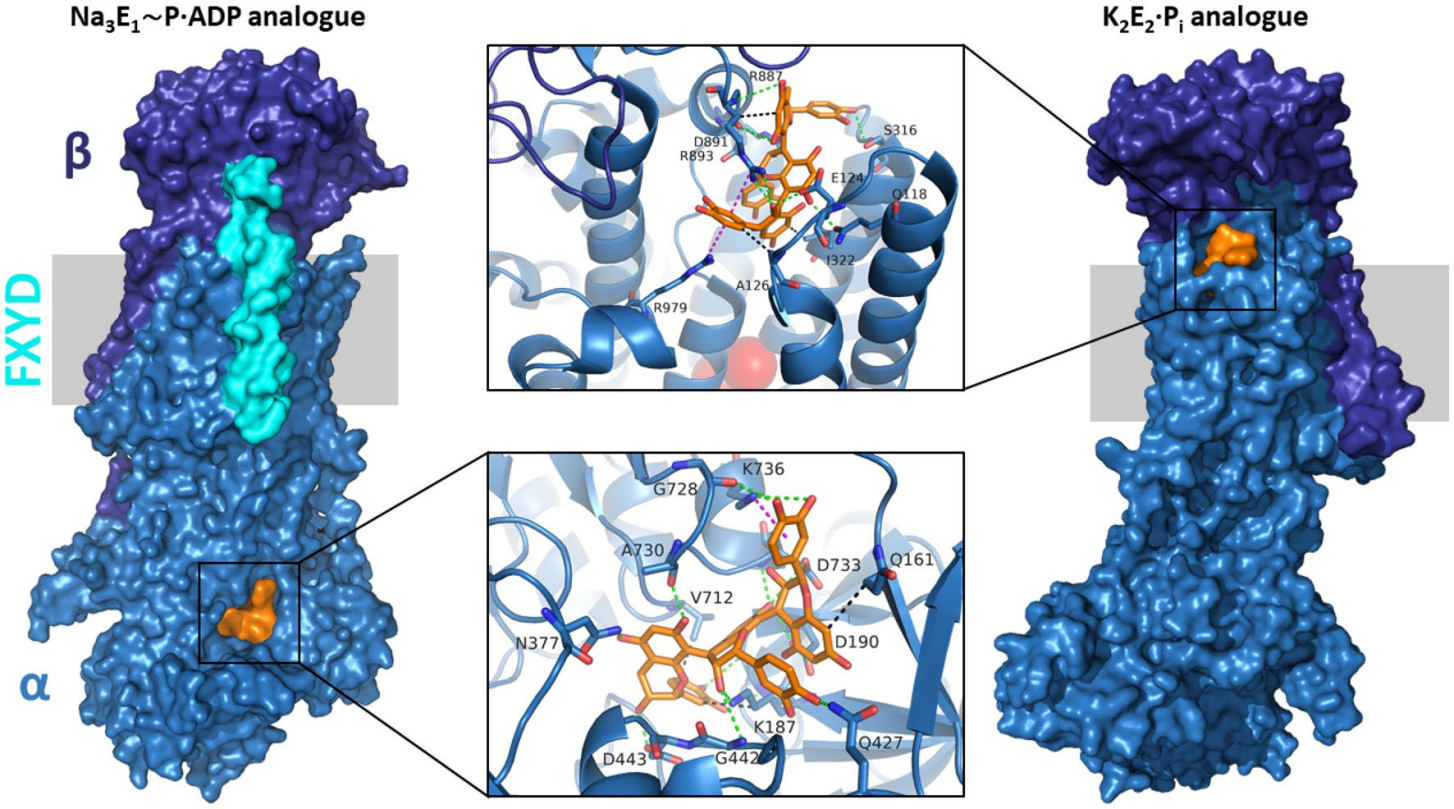
Binding modes of procyanidin C1 in the structures mimicking the Na_3_E_1_∼P·ADP state (left; 3WGU) and the K_2_E_2_·P_i_ state (right; 2ZXE). The membrane region is indicated by the grey area. Surface representation color code: subunit α—sky-blue; subunit β—deep blue; FXYD protein—cyan; procyanidin C1—orange. Non-covalent interactions are shown in the middle using the following color code: hydrophobic—black; hydrogen bonding—green; cation–π—magenta. Potassium ions are represented as red spheres. Figure was created using Avogadro (Avogadro: an open- source molecular builder and visualization tool. Version 1.2. http://avogadro.cc/; 10.1186/1758-2946-4-17).

Docking of procyanidin C1 into the structural analogue of the NKA of Na_3_E_1_∼P·ADP state (3WGU) yielded lower binding energies than those for docking into the K_2_E_2_·P_i_ state analogue. The binding site with the highest affinity for procyanidin C1 (binding energy: –11.5 kcal/mol) was located in close proximity to the nucleotide binding site (Fig. 4, left). For the K_2_E_2_·P_i_ state analogue (2ZXE), the lowest energy binding site of procyanidin C1 (binding energy: –9.9 kcal/mol) was in the extracellular part of the protein (Fig. 4, right). In this case, key protein–ligand interactions included two cation–π bonds between arginine guanidinium groups and the *ortho*-dihydroxyphenyl rings of procyanidin C1.

## Discussion

We used a modern approach based on correlation metabolomics to identify active compounds in a plant extract. This procedure led us to a few candidate compounds and ultimately to the root extract constituent procyanidin C1, which strongly inhibited ATPase activity of NKA (IC_50_ 4.5 µM). In contrast to the strong biological activity of the trimer procyanidin C1, commercially available procyanidin dimers (B1, B2 and B3) were inactive (IC_50_ > 100 µM). The NKA-inhibiting activity of procyanidin C1 was previously unknown, although a procyanidin-rich hawthorn extract WS® 1442 with a procyanidin content of 17.3–20.1% was previously shown to inhibit NKA^58–60^. Potential antiarrhythmic and positive ionotropic effects of WS® 1442 were also reported^62^. However, it remains unclear whether procyanidins or other components of hawthorn extract are primarily responsible for the activity of WS® 1442. For example, ursolic and oleanolic acids, which constitute roughly 0.6% of WS® 1442, are also moderate inhibitors of NKA, with IC_50_ values of 25 to 100 µM^61,62^. Our data on procyanidin C1 suggest that its strong NKA-inhibitory activity together with its high abundance in hawthorn extract may explain the cardiac glycoside-like properties of WS® 1442^63–69^. Procyanidin C1 was also shown to inhibit gastric H^+^,K^+^-ATPase, which is another P-type ATPase with an E_1_–E_2_ catalytic mechanism like NKA. The gastric H^+^,K^+^-ATPase was inhibited by an extract from the aerial part of *Cecropia glaziovii* Snethl. as well as by its chemical constituents procyanidins C1 and B2, whose IC_50_’s were determined to be 46.5 and 40.6 µM, respectively^70^. Due to the presence of other phenolics and the relatively low activity of the identified constituents, the chemical principle of H^+^,K^+^-ATPase inhibition by *Cecropia glaziovii* extract remains unclear.

In this work we investigated the only commercially available trimer, procyanidin C1, which strongly inhibited NKA. Despite its strong activity, procyanidin C1 was present at relatively low concentrations in *V. odorata* when compared to other detected trimers (Fig. 3B),suggesting that they may also contribute to the extract’s activity. It should be noted that the top scoring metabolite given by correlation approach was an unknown trimeric B–type procyanidin, the isomer of procyanidin C1. Due to their similar chemical structures, the procyanidins inevitably coeluted from the SPE cartridges used in this work (C18, MAX and MCX). Therefore, their individual contributions to the overall extract activity are not readily distinguished by correlation alone. However, based on our metabolomic and biochemical data, we suggest that procyanidin C1 is at least partially responsible for the observed activity of *V. odorata* root extracts, acting most likely in combination with its isomer(s). Contributions of extract components other than procyanidin metabolites is unlikely because the only non-procyanidin compound correlated with NKA activity was annotated as feruloyl putrescine, a mono-conjugated phenolamide present in many species that have never been reported to inhibit NKA, including *Salsola subaphylla*, *Zea mays*, *Oryza sativa*, and *Nicotiana tabacum*^71–73^*.* It should also be noted that the presence of procyanidins in the root, the only active *V. odorata* part, might be associated with their role in the nitrogen cycle in the soil^74,75^.

In contrast to the strongly active trimeric procyanidin C1, the dimeric procyanidins B1, B2 and B3 were completely inactive towards NKA. Possible binding modes of procyanidin C1 were identified by molecular docking using the 3WGU and 2ZXE crystal structures of NKA proteins originating from *Sus scrofa* and *Squalus acanthias*, respectively. Both of these structures are available at considerably higher resolutions than other published NKA structures and were therefore the only Na_3_E_1_∼P·ADP and K_2_E_2_·P_i_ state analogues investigated in this work. Comparing the docking results for procyanidin B1, B2, B3, and C1, there is no specific binding site for procyanidin C1 exclusively. Therefore, the three catechin units in procyanidin C1 might be necessary to fulfil spatial requirements for the inhibitory effect. Two interesting bifacial cation–π interactions between arginine guanidinium groups and the *ortho*-dihydroxyphenyl rings of procyanidin C1 were found in the structure mimicking K_2_E_2_·P_i_ state. This interaction causes the ion pathway (which is formed by residues including one of the interacting arginines, R979) to become inaccessible from the extracellular side^76^. This binding mode may thus sterically impede ion exchange. It should be noted that residues R979 in loop L9-10 and D128 in loop L1-2 form a salt bridge in the E_2_P state, but separation of these residues is believed to be required for the motion of the TM2 transmembrane helix during the catalytic cycle^77,78^. Other residues from L1-2 are also important for the protein’s translocase function, so their interactions with the ligand could have additional effects on its conformational transitions^77^. In the best docking pose of the Na_3_E_1_∼P·ADP-mimicking structure (3WGU), the ligand plugged the whole hydrophilic cavity in front of the nucleotide-binding site in the cleft between the nucleotide-binding (N) and actuator (A) domains. In this case, the binding energy (–11.5 kcal/mol) was lower than that for the previously discussed 2ZXE structure (–9.9 kcal/mol). Due to the interdomain location of the binding site, residues from all three cytoplasmic domains are involved in procyanidin C1 binding. The interdomain space in the intracellular part of the protein was previously suggested to be a possible binding site for flavonolignans based on a docking study^79^. A subsequent fluorescence spectroscopy experiment proved that flavonolignans interact with the cytoplasmic segment connecting transmembrane helices TM4 and TM5, providing experimental evidence for a binding mode that may contribute to the inhibition of NKA by flavonolignans^79^. Therefore, our identification of a similarly located binding pose may help explain the inhibitory activity of procyanidin C1.

Procyanidins are highly abundant in human diet, with an estimated daily intake of 57.7 mg per person^80^. On the basis of cell monolayer penetration studies, they are believed to undergo paracellular absorption from the intestine^81–83^. In vivo animal and human studies have also shown that procyanidin dimers and trimers are stable under gastric conditions, are not degraded into monomers, and can be detected in rat plasma and urine after ingestion^84–86,86–88,88–91^. While quantitative data on the plasma concentrations of procyanidins in humans are limited, procyanidin B2 was detected in plasma at a concentration of 4.0 ± 0.6 nM after the consumption of 1.8 mg dimeric procyanidins per kg of body weight. Interestingly, treatment of the plasma samples with sulfatases and β-glucuronidases did not increase the measured procyanidin B2 concentration, suggesting that it was not conjugated^90^. Procyanidins were also detected in human plasma from individuals who had recently consumed procyanidin-rich plant foods/extracts. For example, administration of 0.375 g of cocoa per kg of body weight and 2 g of proanthocyanidin-rich grape seed extract per person resulted in the detection of procyanidins B2 at 41 ± 4 nM and B1 at 10.6 ± 2.5 nM in human plasma^92,93^. Ingested procyanidins thus seem to be readily absorbed in humans, resulting in detectable levels in human plasma. As such, they are both dietary compounds capable of inhibiting NKA and also potential sources of novel molecular structures that could be used as alternatives to cardiac glycosides in the treatment of congestive heart failure and cardiac arrhythmia.

## Conclusions

Procyanidin C1 is a newly discovered NKA inhibitor whose molecular architecture could potentially be optimized to develop analogues with greater druglikeness. Its low micromolar IC_50_ makes all trimeric B-type procyanidins interesting targets for further mechanistic investigation and analysis of structure–activity relationships. Such studies could enable the development of novel NKA inhibitors with previously unexplored modes of action and/or binding sites. The successful application of correlation metabolomics in this work further demonstrates the high effectivity of this approach for identifying biologically active compounds in complex extracts.

## Materials and methods

### Chemicals

Procyanidin B1, B2, B3 and C1 standards (catalog numbers 89764, 89,552, 84,047 and 89,537, respectively) were purchased from PhytoLab (Germany). Stock solutions were prepared by dissolving procyanidins in 50% methanol to a final concentration of 10 mM and stored at –80 °C. The NKA inhibitor ouabain was purchased from Merck (Germany). All solutions were prepared using Milli-Q water with a resistivity above 18.2 MΩ·cm (25 °C).

### Isolation of Na^+^,K^+^-ATPase

NKA was isolated using the protocol of Fedosova^94^ (Supplementary Fig. S1 online), with the following modifications: excess buffer I was poured out from the dissected outer medulla, and the soaked tissue was transferred into a blender. The outer medulla was then homogenized in buffer II with a tissue : buffer ratio of 1 : 3 while ¾ of buffer II was in the form of ice cubes. Aliquots of SDS-treated NKA isolate in buffer II were stored at –80 °C, and were determined to have a protein concentration of 0.44 mg/mL by the Lowry method.

### Na^+^,K^+^-ATPase (NKA) activity assay

Authentic standards (procyanidins B1, B2, B3, and C1; ( +)-catechin and (–)-epicatechin) dissolved in 50% methanol with a stock concentration of 10 mM were diluted to a final concentration of 100 µM for use in assays. Crude extracts of *Viola odorata* L. seed, root, and leaf with concentration of 240 mg DW/mL in dimethyl sulfoxide were diluted to a final concentration of 600 µg DW/mL. Fractions of crude *V. odorata* L. root extract with stock concentrations of 240 mg DW/mL in 20% methanol with 0.1% HCOOH were diluted to a final concentration of 480 µg DW/mL.

Inorganic phosphate (P_i_) quantification was conducted in microplates using a previously reported method^79^. Each ATPase reaction was performed in four wells without ouabain to determine total liberated P_i_ and in four wells with ouabain to quantify P_i_ not generated by ouabain-sensitive NKA activity.

The ouabain-sensitive ATPase activity of NKA (henceforth referred to as NKA activity) was measured by adding 30 µL of a 1.67 × concentrated buffered NKA solution to 10 µL of pre-concentrated inhibitor solution diluted in water. NKA was pre-incubated with the inhibitor for 5 min, then the ATPase reaction was started by adding 10 µL of 15 mM Na_2_ATP dissolved in 25 mM Tris base. The final buffered (pH 7.2) NKA solution contained 4 mM MgCl_2_, 20 mM KCl, 130 mM NaCl, 30 mM l-histidine, and 26 µg/mL of NKA protein with or without 1 mM ouabain. The ATPase reaction lasted for 6 min after which 75 µL of solution II was added to develop color (2.9% (w/v) sodium dodecyl sulfate, 0.5% (w/v) ammonium heptamolybdate tetrahydrate, 163 mM ascorbic acid, and 3.7% (w/v) HCl). After 8 min, color development was stopped by adding 125 µL of solution III (3.5% (w/v) bismuth citrate, 3.5% (w/v) trisodium citrate dihydrate, 3.7% (w/v) HCl). Finally, the absorbance was measured at 710 nm using an Infinite 200 microplate reader (Tecan, Switzerland).

The NKA activity observed in the presence of each inhibitor was normalized against a control activity determined by mixing the NKA solution with water instead of an inhibitor solution in order to obtain the relative NKA activity (%) for each inhibitor. NKA activity was calculated by subtracting the average absorbance of ouabain containing samples from that of samples without ouabain. Dose–response curves were generated by using a four-parameter logistic function (fixed 0–100% range) to fit the experimental data.

### Plant collection and extraction

*Viola odorata* L., collected in Bělkovice Valley (Olomouc Region, Czech Republic), was identified by Michal Hroneš, Ph.D. (Department of Botany, Palacký University Olomouc). The collection complied with all applicable laws/guidelines, both institutional and national. According to IUCN guidelines, this taxon is not threatened being qualified for Least Concern category. As a common plant species, it’s collection for scientific purpose requires no permission/license. Plants were freeze-dried and homogenized using blade grinder. 30 mg of dried plant material (DW) was extracted in a microtube using 1 mL of 0.1% HCOOH in methanol. Glass balls were added to the microtube and the plant material was homogenized for 5 min using an MM 400 oscillatory ball mill (RETSCH, Germany) operating at 27 Hz. An ultrasound-assisted extraction (10 min, 45 kHz) in a USC100T ultrasonic bath (VWR, USA) and subsequent centrifugation (21,300 g, 25 °C, 10 min) followed. The supernatant was collected and evaporated at 37 °C in a TurboVap Classic LV nitrogen evaporator (Biotage, Sweden).

### Plant extract fractionation

Methanol, aqueous ammonia and HCOOH used for fractionation were of LC–MS grade.

#### Reverse phase C18 SPE fractionation

A solid-phase extraction (SPE) C18 sorbent cartridge (*Spe-ed* 6 mL octadecyl/18; Applied Separations, USA) was conditioned with 12 mL of methanol and equilibrated with 2 mL of 0.1% HCOOH. Evaporated crude *Viola odorata* root extract (24 mg DW) dissolved in 2 mL of 0.1% HCOOH was loaded onto the column. Fractions RP0, RP1, RP2, RP3, RP4, RP5, RP6, and RP7 were obtained by eluting the crude extract from the sorbent with 2 mL of 0.1% HCOOH in aqueous methanol of gradually increasing concentration (0, 10, 20, 30, 50, 60, 70, and 100%).

#### Mixed-mode anion exchange SPE fractionation

A mixed-mode anion exchange (MAX) SPE sorbent cartridge (Oasis MAX, 150 mg, 6 mL; Waters, USA) was conditioned with 12 mL of methanol and equilibrated first with 6 mL of water followed by 6 mL of 5% aqueous ammonia. Evaporated crude *V. odorata* root extract (24 mg DW) dissolved in 5% aqueous ammonia was loaded onto the column and washed with 5 mL of the loading solvent (eluting fraction MA0). Fractions obtained by elution with 4 mL of methanol (MA1) and 4 mL of 2% methanolic HCOOH (MA2) were then collected.

#### Mixed-mode cation exchange SPE fractionation

 A mixed-mode cation exchange (MCX) SPE sorbent cartridge (Oasis MCX, 150 mg, 6 mL; Waters, USA) was conditioned with 12 mL of methanol and equilibrated firstly with 6 mL of water, then with 6 mL of 2% aqueous HCOOH. Evaporated crude *V. odorata* root extract (24 mg DW) dissolved in 2% aqueous HCOOH was loaded onto the column and washed with 5 mL of loading solvent (eluting fraction MC0). Fractions obtained by elution with 4 mL of methanol (MC1) and 4 mL of 5% methanolic ammonia (MC2) were collected.

Each collected fraction was evaporated at 37 °C in a nitrogen evaporator and then redissolved in 100 µL of 20% methanol with 0.1% HCOOH. Aliquots were taken for use in LC–MS analyses and NKA inhibition assays.

### UHPLC-QTOF-MS/MS

UHPLC-QTOF-MS/MS analyses were performed as described previously^56^. Briefly, samples dissolved in 20% methanol with 0.1% HCOOH were filtered through a 0.2 μm regenerated cellulose membrane microfilter (Grace, USA) prior to UHPLC analysis. 5 µL of each sample was injected onto a reversed-phase column (BEH C18, 1.7 μm, 2.1 × 150 mm, Waters, USA) maintained at 30 °C. Elution was performed over 22 min using the following binary gradient of acetonitrile (A) and 5 mM aqueous HCOOH (B): 0 min 5% A, 1 min 10% A, 12 min 35% A, 17 min 70% A, 17.5 min 100% A. The nebulizer gas pressure was set to 6 bar, desolvation temperature to 500 °C, and desolvation gas flow to 600 L/h. Analytes were ionized using an ESI source (120 °C, capillary 2 kV, cone voltage 25 V, cone gas flow 30 L/h) operating in both negative and positive ion modes. MS data were recorded in the *m/z* range of 70 − 1,500. MS/MS experiments were performed using a collision energy of 20 eV.

In-house developed MATLAB algorithms were applied to raw LC–MS data generated by MassLynx software to produce a list of features characterized by retention time, *m/z*, and peak area. Only features with retention times between 2 and 18 min and a peak area above 5,000 AU in at least one sample were considered. Pearson correlation coefficients were computed between feature area and inhibitory activity (expressed in µmol of ouabain equivalents per 1 g of DW). Features with the highest correlation coefficients were manually processed using Masslynx 4.1 software (Waters, USA) to identify candidate pseudo-molecular ions, their elemental compositions, and the corresponding mass accuracy. Procyanidins B1, B2, B3 and C1 were identified based on their retention times and comparisons to the fragmentation patterns of authentic standards.

### Molecular docking

The structures of procyanidins B1, B2,B3 and C1 obtained from the PubChem database was optimized using Avogadro (Avogadro: an open-source molecular builder and visualization tool. Version 1.2. http://avogadro.cc/; 10.1186/1758-2946-4-17) using the energy minimizing tool with the General Amber Force Field. NKA crystal structures (PDB ID 3WGU, 2.80 Å109 and 2ZXE, 2.40 Å110) were downloaded from the Protein Data Bank (www.rcsb.org). Ligands and protein structures without waters, ions, and other ligands were prepared for docking using Autodock Tools (10.1002/jcc.21256, version 1.5.6). Ligand bonds whose rotation generated different conformers were allowed to rotate freely in accordance with the software’s default settings. Docking was performed using Autodock Vina^95^, with a grid box covering the whole protein. Exhaustiveness was set to 100 and available 20 binding modes were obtained. Redocking was performed with grid box limited to frequently occupied binding sites.

Protein–ligand interactions of redocked binding modes were analyzed using the Protein–Ligand Interaction Profiler (PLIP)^96^. Residue numbering remained the same as in the original crystal structures. Figures for publication were prepared using PyMOL (The PyMOL Molecular Graphics System, Version 2.4.1, Schrödinger, LLC.).

### Statistical analysis

All statistical analyses were performed using GraphPad Prism 8.0 software (GraphPad Software, Inc; USA). Data on NKA activity were analyzed by one-way ANOVA followed by Tukey’s post hoc test. Values are given as mean ± SD. Statistical significance is reported as **** *p* < 0.0001, *** *p* < 0.001, ** *p* < 0.01 and * *p* < 0.05.

## Supplementary Information

Supplementary Information.

## Data Availability

The datasets generated during and/or analysed during the current study are available from the corresponding author on reasonable request.
