# Supplementary material for: Procyanidin C1 from Viola odorata L. inhibits Na+,K+-ATPase
Source: Sci Rep. 2022 Apr 29;12:7011. doi: 10.1038/s41598-022-11086-y (PMC9055044; doi:10.1038/s41598-022-11086-y)
Supplement: Supplementary file 1 — Supplementary Information. [file 41598_2022_11086_MOESM1_ESM.pdf]

Supplementary information:

Title:

Procyanidin C1 from *Viola odorata* L. inhibits Na^+,K^+-ATPase

Authors:

Tomas Heger[1], Marek Zatloukal[2], Martin Kubala[3], Miroslav Strnad[4] and Jiri Gruz[1,*]

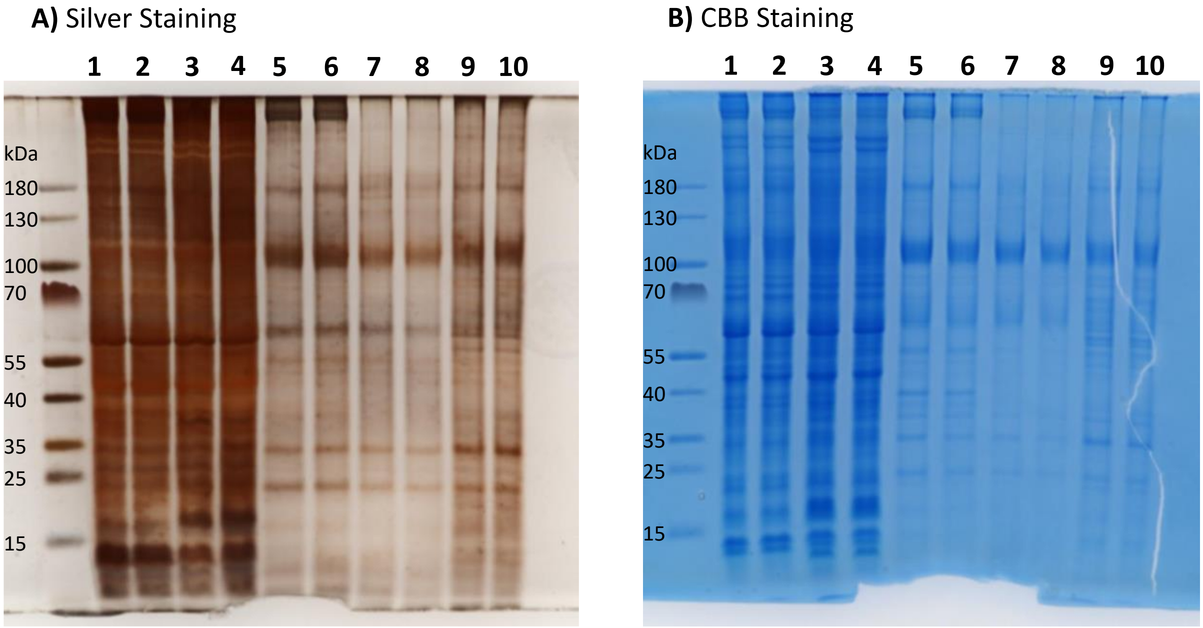

Figure S1. SDS-PAGE gels stained by silver or CBB (Coomassie Brilliant Blue G-250). Lines 1, 2 – sample of the microsomal fraction from the preparation A (34.5 µg protein, duplicate). Lines 3, 4 – sample of the microsomal fraction from the preparation B (34.5 µg protein, duplicate). Lines 5, 6 – sample of the SDS-treated NKA fraction from the preparation A (7.5 µg protein, duplicate). Lines 7, 8 – sample of the SDS-treated NKA fraction from the preparation C (7.5 µg protein, duplicate). Lines 9, 10 – sample of the SDS-treated NKA fraction from the preparation B (7.5 µg protein, duplicate). In this study, the SDS-treated NKA fraction from the preparation B was used for the NKA activity assays.

Table S1. List of plant species screened for NKA inhibiting activity.

Plant Species	Plant Part	NKA inhibition (%)	SD
Incarvillea olgae Regel	leaf	77.6	2.4
Hyssopus officinalis L.	leaf & stem	86.0	11.2
Hyssopus officinalis L.	flower	94.2	6.1
Deutzia corymbosa R.Br. ex G.Don	leaf	49.3	1.8
Platycodon grandiflorus (Jacq.) A.DC.	leaf	73.2	2.5
Mercurialis annua L.	aerial part	98.6	4.1
Mercurialis annua L.	root	98.1	7.4
Asplenium trichomanes L.	leaf	60.7	6.6
Perilla frutescens var. crispa (Benth.) W.Deane	leaf	156.2	13.5
Solanum sisymbriifolium Lam.	root	80.9	16.3
Solanum sisymbriifolium Lam.	fruit	78.9	24.2
Solanum sisymbriifolium Lam.	leaf	80.0	21.8
Lonicera henryi Hemsl.	leaf	71.7	21.2
Nepeta sibirica L.	leaf	54.7	10.8
Ophiopogon planiscapus 'Nigrescens' Nakai	leaf	75.6	22.0
Cotula hispida (DC.) Harv.	leaf & stem	145.8	12.8
Leptinella squalida Hook.f.	leaf & stem	114.3	36.8
Leucophyta brownii Cass.	leaf & stem	78.2	12.8
Aubrieta deltoidea 'Cascade Blue' (L.) DC.	leaf & stem	68.3	7.3
Lewisia pygmaea (Gray) B.L.Rob.	leaf	67.7	7.4
Acanthus mollis L.	leaf	61.5	10.7
Coreopsis verticillata 'Red Ruby' L.	flower	22.4	7.6
Veronica rakaiensis J.B.Armstr.	leaf & stem	133.5	5.8
Erythrina crista-galli L.	leaf	99.7	8.6
Kniphofia uvaria 'Alcazar' (L.) Oken	flower	116.7	10.8
Kniphofia uvaria 'Alcazar' (L.) Oken	flower stalk	114.8	13.1
Kniphofia uvaria 'Alcazar' (L.) Oken	leaf	120.2	3.4
Hosta sp. Tratt.	flower	90.3	4.0
Hosta sp. Tratt.	leaf	81.8	11.2
Teucrium chamaedrys L.	flower	100.4	3.2
Teucrium chamaedrys L.	leaf	103.7	11.9
Glechoma hederacea L.	aerial part	77.1	7.3
Tribulus terrestris L.	fruit	120.8	8.6
Veronica spicata L.	flower	121.0	14.1
Veronica spicata L.	leaf	91.6	28.3
Tradescantia virginiana L.	leaf	124.5	6.7
Ruta graveolens L.	leaf	100.6	9.5
Linaria vulgaris Mill.	aerial part	130.2	22.4
Salvia transsylvanica Schur	flower	119.6	1.4
Salvia transsylvanica Schur	leaf	124.0	3.8
Betonica officinalis L.	flower	104.7	21.0
Betonica officinalis L.	leaf	103.0	6.3
Stachys recta L.	leaf	85.4	1.4
Verbena officinalis L.	aerial part	103.7	12.4
Solidago virgaurea L.	flower	96.4	22.3
Melilotus officinalis (L.) Lam.	aerial part	108.0	16.0
Euphrasia officinalis L.	leaf & flower	118.7	7.1
Silene flos-cuculi (L.) Greuter & Burdet	flower	120.4	9.1
Silene flos-cuculi (L.) Greuter & Burdet	leaf	125.8	4.9
Viscaria vulgaris Bernh.	flower	78.7	6.4
Viscaria vulgaris Bernh.	leaf	113.5	9.3
Campanula glomerata L.	leaf	102.0	13.4
Viola tricolor L.	root	99.7	8.5
Viola tricolor L.	flower	119.7	6.1

Viola tricolor L.	leaf	114.4	15.6
Viola philippica Cav.	leaf	99.5	11.3
Viola philippica Cav.	root	70.6	4.8
Pilosella aurantiaca (L.) F.W.Schultz & Sch.Bip.	leaf	119.8	9.0
Corydalis solida (L.) Clairv.	aerial part	124.9	10.3
Corydalis solida (L.) Clairv.	root	123.5	8.4
Euterpe oleracea Mart.	fruit	96.4	8.0
Aloe vera (L.) Burm.f.	leaf	108.7	8.4
Hippophae rhamnoides L.	fruit	111.8	9.0
Triticum aestivum L.	sprout	82.3	15.8
Brassica rapa subsp. Pekinensis (Lour.) Hanelt ex Mansf.	leaf	119.3	18.4
Petroselinum crispum (Mill.) Fuss	leaf & stem	121.1	11.2
Lavandula angustifolia Mill.	flower	91.6	10.8
Lavandula angustifolia Mill.	leaf	96.4	6.1
Callisia fragrans (Lindl.) Woodson	leaf	111.4	2.1
Tropaeolum majus L.	fruit	130.3	10.2
